# Supplementary material for: First Genome Description of Providencia vermicola Isolate Bearing NDM-1 from Blood Culture
Source: Microorganisms. 2021 Aug 17;9(8):1751. doi: 10.3390/microorganisms9081751 (PMC8398168; doi:10.3390/microorganisms9081751)
Supplement: Supplementary file 1 [file microorganisms-09-01751-s001.zip › Supplementary Table S1.pdf]

Supplementary Table S1: BlastP comparison of the Siderophore biosynthesis NRPS cluster of *P. vermicola* P8538 with that of *P. stuartii*, *M. morganii*, and *Salmonella enterica.*

***P. vermicola* P8538**	Size (aa)	%GC content	Predicted functions	***Providencia stuartii* PRV00010**		***Morganella morganii* VGH116**		***Salmonella enterica* 2014AM-3158**	
				**%aa identity**	**E-value**	**%aa identity**	**E-value**	**%aa identity**	**E-value**
ORF1	106	32.09	Helix-turn-helix transcriptional regulator	100	1.09e-77	0	-	0	-
ORF2	207	40.22	Fimbrial protein	100	2.02e-126	0	-	0	-
ORF3	227	33.63	Hypothetical protein	99	3.24e-142	0	-	0	-
ORF4	831	36.14	CS1-pili formation C-terminal domain-protein	100	0.0	0	-	0	-
ORF5	515	35.98	CFA/I fimbrial minor adhesin	100	0.0	0	-	0	-
ORF6	213	32.24	Fimbrial protein	100	1.07e-159	0	-	0	-
ORF7	246	40.76	Glycerophosphodiester phosphodiesterase	100	3.55e-162	0	-	0	-
ORF8	239	33.19	4'-phosphopantetheinyl transferase entD	100	2.22e-173	46	5.67e-75	0	-
ORF9	673	36.75	TonB-dependent receptor	100	0.0	72	0.0	69	0.0
ORF10	1498	30.11	Amino acid adenylation domain-protein	99	0.0	52	0.0	52	0.0
ORF11	2628	30.25	**Siderophore** biosynthesis NRPS modules (AMP-binding protein)	99	0.0	56	0.0	56	0.0
ORF12	1008	35.81	Acyltransferase domain-containing protein (Type I polyketide synthase)	100	0.0	64	0.0	64	0.0
ORF13	73	26.58	Acyl carrier protein	100	8.51e-50	64	1.51e-27	60	4.34e-27
ORF14	530	30.13	2.3-dihydroxybenzoyl)adenylate synthase (AMP-binding protein)	98	0.0	65	0.0	66	0.0
ORF15	414	28.51	Non-ribosomal peptide synthetase	99	0.0	54	4.69e-166	53	2.66e-164
ORF16	1438	30.14	Thioester reductase domain-containing protein (AMP-binding protein)	99	0.0	58	0.0	58	0.0
ORF17	257	33.33	Thioesterase	99	0.0	66	5.81e-120	66	3.00e-125
ORF18	585	32.37	ABC transporter ATP-binding protein	100	0.0	77	0.0	77	0.0
ORF19	585	34.19	ABC transporter ATP-binding protein	100	0.0	79	0.0	80	0.0
ORF20	415	33.17	Tetracycline resistance MFS efflux pump	99	0.0	79	0.0	79	0.0
ORF21	403	32.43	MFS transporter	100	0.0	74	0.0	72	1.06e-147
ORF22	497	33.53	Aminotransferase class V-fold PLP-dependent enzyme	100	0.0	68	0.0		
ORF23	449	34.15	Aromatic acid/H+ symport family MFS transporter	98	0.0	62	8.32e-158	29	3.40e-05

ORF24	247	43.28	2,3-dihydro-2,3-dihydroxybenzoate dehydrogenase	99	0.0	55	1.75e-94	0	-
ORF25	218	43.38	Isochorismatase family protein	100	6.02e-168	61	2.19e-97	0	-
ORF26	383	43.14	Isochorismate synthase	99	0.0	46	4.77e-113	0	-
ORF27	119	40.28	Hypothetical protein	0	-	0	-	0	-
