# Supplementary material for: First Genome Description of Providencia vermicola Isolate Bearing NDM-1 from Blood Culture
Source: Microorganisms. 2021 Aug 17;9(8):1751. doi: 10.3390/microorganisms9081751 (PMC8398168; doi:10.3390/microorganisms9081751)
Supplement: Supplementary file 1 [file microorganisms-09-01751-s001.zip › Supplementary Table S2.pdf]

Supplementary Table S2: Virulence factor genes identified in ***Providencia vermicola* P8538** isolate.

Gene	Description	Best Blast Hit	Identity (%)	Coverage (%)	Accession Number
hcp/tssD	Type VI secretion system protein, Hcp family	*Klebsiella pneumoniae* HS11286	77.24	100	YP_005226602.1
gnd	6-phosphogluconate dehydrogenase	*Klebsiella pneumoniae* NTUH-K2044	76.17	100	YP_002920353.1
acrB	Acriflavine resistance protein B	*Klebsiella pneumoniae* NTUH-K2044	70.49	99.34	YP_002918165.1
kdsA	2-dehydro-3-deoxyphosphooctonate aldolase	*Haemophilus influenzae* Rd KW20	74.82	98.83	NP_439706
rfaD	ADP-L-glycero-D-mannoheptose-6-epimerase	*Haemophilus influenzae* Rd KW20	72.58	98.83	NP_439271
htpB	Hsp60, 60K heat shock protein Htpb	*Legionella pneumophila* 1	73.95	98.72	YP_094724
fliG	Flagellar motor switch protein G	*Yersinia enterocolitica* 8081	71.46	98.59	YP_001006742
cheY	Chemotaxis regulatory protein Chey	*Yersinia enterocolitica* 8081	75.78	97.46	YP_001006774
vipB/tssC	Type VI secretion system contractile sheath large subunit Vipb	*Klebsiella pneumoniae* HS11286	70.59	95.45	YP_005226598.1
lpxC	UDP-3-O-(R-3-hydroxymyristoyl)-N-acetylglucosamine deacetylase	*Haemophilus influenzae* Rd KW20	73.91	94.99	NP_439302
rcsB	Transcriptional regulator Rcsb	*Klebsiella pneumoniae* NTUH-K2044	73.53	94.01	YP_002920501.1
flhC	Flagellar biosynthesis transcription activator Flhc	*Yersinia enterocolitica* 8081	75	90.82	YP_001006783
fliM	Flagellar motor switch protein Flim	*Yersinia enterocolitica* 8081	70.08	87.8	YP_001006748
fliQ	Flagellar biosynthetic protein Fliq	*Yersinia enterocolitica* 8081	71.37	86.3	YP_001006752
fliI	Flagellum-specific ATP synthase Flii	*Yersinia enterocolitica* 8081	71.92	84.65	YP_001006744
flgG	Flagellar basal-body rod protein Flgg	*Yersinia enterocolitica* 8081	71.08	83.52	YP_001006759
cheW	Purine-binding chemotaxis protein Chew	*Yersinia enterocolitica* 8081	71.01	82.53	YP_001006779
luxS	S-ribosylhomocysteinase	*Vibrio cholerae O1 biovar El Tor* N16961	72.42	80.62	NP_230208
flhA	Flagellar biosynthesis protein Flha	*Yersinia enterocolitica* 8081	71.02	73.82	YP_001006770
gmhA/lpcA	Phosphoheptose isomerase	*Haemophilus influenzae* Rd KW20	77.97	71.33	NP_439337
