# Supplementary figures and images for: First Genome Description of Providencia vermicola Isolate Bearing NDM-1 from Blood Culture
Source: Microorganisms. 2021 Aug 17;9(8):1751. doi: 10.3390/microorganisms9081751 (PMC8398168; doi:10.3390/microorganisms9081751)

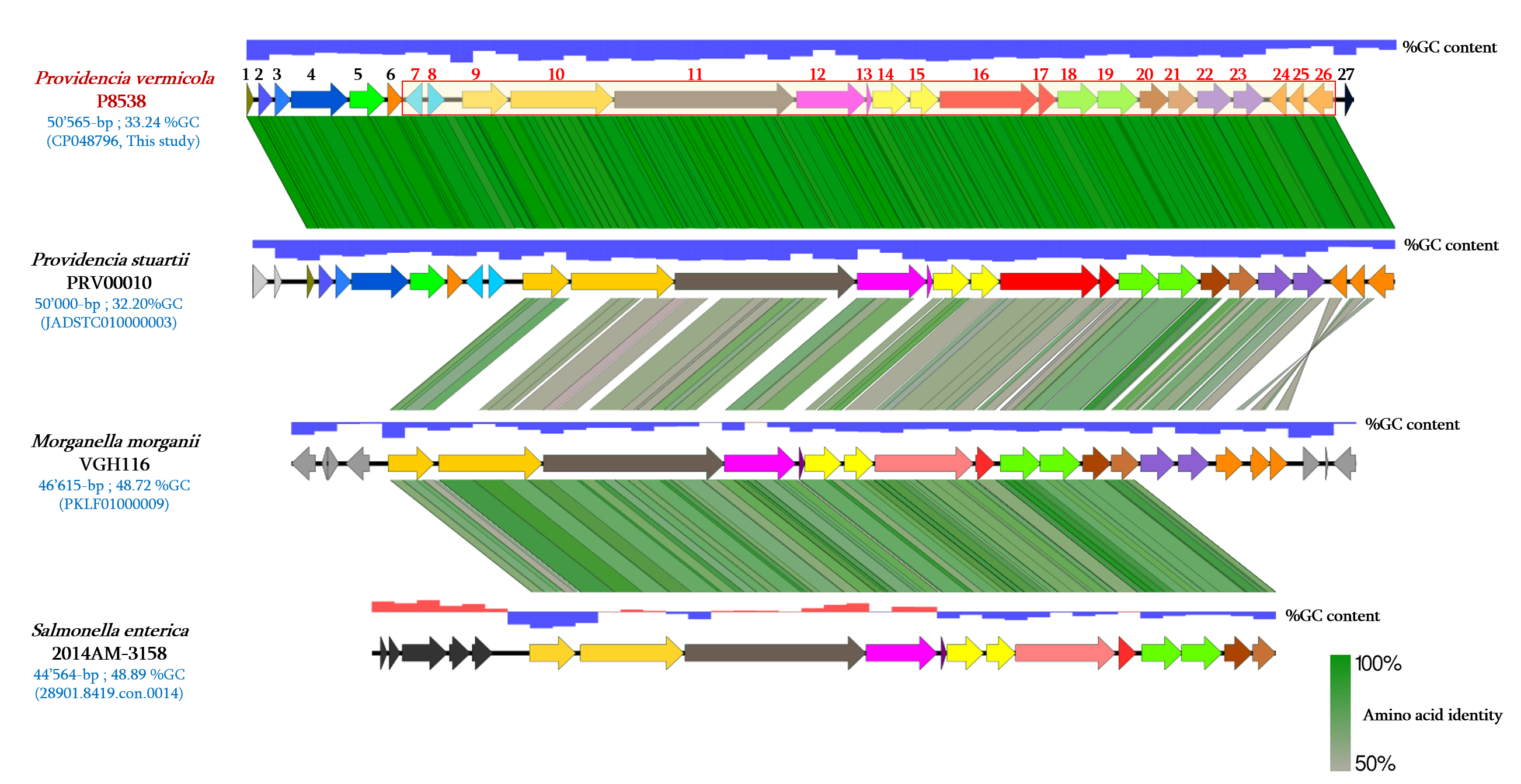

Supplement: Supplementary file 1 [file microorganisms-09-01751-s001.zip › Suppl. Figure S1.tif]
